# Meningioma Radiomics: At the Nexus of Imaging, Pathology and Biomolecular Characterization

**DOI:** 10.3390/cancers14112605

**Published:** 2022-05-25

**Authors:** Lorenzo Ugga, Gaia Spadarella, Lorenzo Pinto, Renato Cuocolo, Arturo Brunetti

**Affiliations:** 1 Department of Advanced Biomedical Sciences, University of Naples “Federico II”, Via Sergio Pansini 5, 80131 Naples, Italy; gaia.spadarella@gmail.com (G.S.); lorenzo.pinto@unina.it (L.P.); brunetti@unina.it (A.B.); 2 Department of Medicine, Surgery and Dentistry, University of Salerno, Via Salvador Allende 43, 84081 Baronissi, Italy; renato.cuocolo@unina.it

**Keywords:** meningioma, radiomics, diagnostic imaging, artificial intelligence

## Abstract

**Simple Summary:**

Meningiomas are typically benign, common extra-axial tumors of the central nervous system. Routine clinical assessment by radiologists presents some limitations regarding long-term patient outcome prediction and risk stratification. Given the exponential growth of interest in radiomics and artificial intelligence in medical imaging, numerous studies have evaluated the potential of these tools in the setting of meningioma imaging. These were aimed at the development of reliable and reproducible models based on quantitative data. Although several limitations have yet to be overcome for their routine use in clinical practice, their innovative potential is evident. In this review, we present a wide-ranging overview of radiomics and artificial intelligence applications in meningioma imaging.

**Abstract:**

Meningiomas are the most common extra-axial tumors of the central nervous system (CNS). Even though recurrence is uncommon after surgery and most meningiomas are benign, an aggressive behavior may still be exhibited in some cases. Although the diagnosis can be made by radiologists, typically with magnetic resonance imaging, qualitative analysis has some limitations in regard to outcome prediction and risk stratification. The acquisition of this information could help the referring clinician in the decision-making process and selection of the appropriate treatment. Following the increased attention and potential of radiomics and artificial intelligence in the healthcare domain, including oncological imaging, researchers have investigated their use over the years to overcome the current limitations of imaging. The aim of these new tools is the replacement of subjective and, therefore, potentially variable medical image analysis by more objective quantitative data, using computational algorithms. Although radiomics has not yet fully entered clinical practice, its potential for the detection, diagnostic, and prognostic characterization of tumors is evident. In this review, we present a wide-ranging overview of radiomics and artificial intelligence applications in meningioma imaging.

## 1. Background

Meningioma is the most frequent central nervous system (CNS) neoplasm, accounting for 36% of all primary brain tumors [1]. It arises from arachnoid cap cells associated with the dura mater or choroid plexus. Older age and female sex represent demographic factors associated with increased meningioma risk [1]. According to the 2016 World Health Organization (WHO) Classification of Tumors of the CNS, meningiomas are classified into three grading subgroups, ranging from I to III [2]. This grading system has been confirmed, with some minor changes, in the recently published 5th edition of the WHO classification [3]. Most of these neoplasms are WHO grade I, consisting of benign, slowly growing lesions. Atypical meningiomas (WHO grade II) represent 20–25% of cases and show an increased likelihood of recurrence. Finally, 1–6% are classified as WHO grade III, presenting malignant behavior with metastatic potential. Magnetic resonance imaging (MRI) is the modality of choice for assessing these tumors, while computed tomography (CT) can be acquired when MRI cannot be performed or in combination with MRI to better depict dystrophic calcifications and bone changes. In recent years, radiomics applications have shown the potential to provide additional information from medical images of patients affected by meningioma. Clinical implementation of radiomics-based classification and predictive models could be of great impact in the management of these patients in the near future.

In this review, we aim to provide an overview of radiomics studies focused on different research areas of meningioma imaging, such as lesion segmentation, differential diagnosis, and recurrence prediction (Table 1).

## 2. Radiomics and Artificial Intelligence

Radiomics is an emerging field of research, which has gained a great amount of attention in the decade since its formal definition [27]. It consists of a collection of techniques aimed at obtaining quantitative image descriptors, some of which date back several decades [28,29]. Therefore, radiomics analyses are based on the extraction and modeling of high-dimensional, quantitative feature sets derived from regions of interest within medical images. In the current era of precision medicine, this approach presents an enormous potential. The hypothesis justifying the use of radiomics is represented by the correlation of quantifiable image grey level heterogeneity within a lesion and previously established biological, e.g., phenotypical or genotypical, characteristics of interest. If this were proven as true, it would allow for the definition of non-invasive imaging biomarkers that could substitute traditional pathological or genomic lesion profiling. Furthermore, entirely novel radiomics biomarkers could be developed (e.g., radiomics signatures), with prognostic or diagnostic value. Overall, this approach has the potential to uncover tumoral characteristics, not identified by qualitative or traditional quantitative analyses, which could provide crucial information for decision support. Typically, the radiomics workflow starts with image acquisition and preprocessing, followed by lesion segmentation after the which extraction of mineable features can be performed [30].

Compared to other fields of quantitative image analysis, such as brain functional MRI or voxel-based morphometry studies, there is still no consensus on the most appropriate tools or even specific aims of image preprocessing. In general, it is known that differences in scanner vendor or technology, as well as differences in image acquisition parameters does impact the reproducibility of radiomics features [31]. Noise introduced in the features due to these issues is clearly undesirable and will result in biased models. Therefore, the rationale behind the use of image preprocessing techniques is represented by the harmonization of images to reduce variance caused by acquisition protocol differences. In other words, the ideal use case is represented by heterogeneity entirely due to biological characteristics rather than image noise or acquisition parameters. This is clearly unattainable in practice, but denoising techniques as well as grey level normalization and discretization can mitigate these issues. However, the specific software implementations and settings for these tasks are still debated and may even vary based on the specific use case. One of the solutions proposed to address this limitation is represented by domain expert working groups that are developing common reference standards for radiomics features and the entire workflow.

The most well-known and successful of such groups is represented by the Image Biomarker Standardization Initiative (IBSI), which provide guidelines [32], aimed at increasing reproducibility and to allow for the translation of radiomic studies to the clinical setting. This is especially relevant for the extraction process since there are different techniques and formulas that may be implemented to calculate textural descriptors. Radiomics data include morphologic features as well as first-, second-, and higher-order statistics. Morphologic features describe geometric characteristics of the region of interest, such as volume, surface area, compactness, and sphericity. First-order statistics include grey level histogram-derived features, while second-order characteristics describe the spatial variation in pixel intensities (texture features). It should be noted that all previously mentioned groups of features can be obtained not only from the original pre-processed images but also after the application of noise reduction and/or texture-enhancing filters. For example, commonly used filtering tools are wavelet decomposition and Laplacian of Gaussian [33].

Considering the large amount of data to be analyzed, classical statistical approaches are generally not preferable for conducting the entire analysis. On the contrary, data analysis techniques based on artificial intelligence are particularly fitting for this task. Often, radiomics pipeline includes a combination of classical statistics and machine learning algorithms. The first are commonly used for data pre-processing and feature selection, the latter for feature selection and classification or regression modeling. Machine learning tools are a subset of artificial intelligence (AI) that deals with systems that produce models directly based on the data available for their training process. These algorithms have an incredible potential and may also be improved over time by increasing the amount of available training data.

Three main machine learning approaches are commonly described:

- Supervised learning: the algorithm input is provided as a labeled training dataset (ground truth); this is the most commonly employed technique in medical imaging. Supervised learning includes classification and regression algorithms. Classification algorithms aim to assign specific categories to new data instances. Linear classifiers, support vector machines, decision trees, and ensemble methods (e.g., random forest) are common types of classification algorithms. On the other hand, regression algorithms attempt to estimate the mapping function from input variables to continuous output variables.
- Unsupervised learning: the algorithm explores the underlying patterns and predicts the output without a labeled database; for this reason, the post hoc interpretation of the resulting clusters may be very complex, and a large amount of training data is usually required.
- Reinforcement learning: based on feedback loops (negative and/or positive reinforcement) and requires a trial–error process. This approach has been commonly applied in robotics, telecommunications, and game theory fields.

These types of learning can work singularly or in combination (example: semi-supervised learning). Deep learning is a subfield of machine learning which progressively extracts higher-level features from the raw input using multiple layers of data processing nodes, in a hierarchy of increasing complexity and abstraction. It is based on neural networks, whose circuits of interconnected artificial neurons (i.e., nodes) were originally inspired by the human brain’s architecture.

Independently of the type of training and algorithm architecture, any model obtained from radiomics data should undergo thorough validation. There are several strategies that allow for better estimation of model performance on new data, such as resampling the data through k-fold cross-validation. In this approach, the training data is split in k folds which are iteratively employed for model training (k-1 folds of data) with the final subset for validation. The resulting mean accuracy metrics are more robust to variations in performance due to the random split of the data in training and validation sets, especially if the entire process is repeated multiple times. However, an external validation of the final model is also crucial. This can be more informative of the actual performance in a clinical setting, even though the limited availability of medical imaging data still represents a significant limitation compared to other domains. Particular care should also be taken to distinguish analyses performed to compare and tune different hyperparameters of a radiomics pipeline and those that assess the overall accuracy of the resulting model on new data. Both these processes may be referred to as validation or testing, based on the study design and common practice in the research group’s reference domain (e.g., healthcare, machine learning, statistics), but the interpretation of the resulting accuracy metrics should be appropriately presented to the general readership.

## 3. Lesion Segmentation

**Figure 1:**
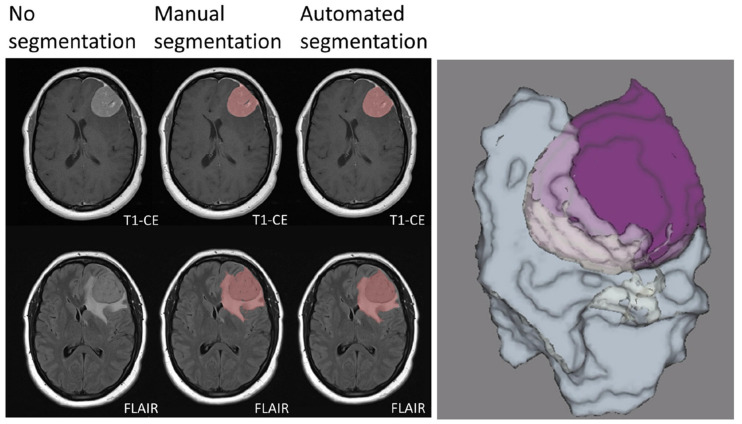
Manual and automated segmentation comparison in a meningioma of the left frontal convexity. The sharply demarcated lesion demonstrates intense contrast enhancement. Vasogenic edema of the surrounding white matter is also evident. Manual and automated segmentation are correctly matched. Three-dimensional rendering of the segmented tumor and edema volumes is also presented. T1-CE: contrast-enhanced T1-weighted imaging; FLAIR: fluid attenuated inversion recovery. Adapted from Ref. [13], under the terms of the Creative Commons Attribution-NonCommercial 4.0 License.

Segmentation represents a critical step in the radiomics workflow as features are extracted from the segmented regions of interest (ROI). Furthermore, in the context of meningiomas, volumetric assessment may also be relevant for therapy planning and monitoring. While manual segmentation, although more accurate, is time consuming and prone to inter-observer variability, semi-automated and fully automated approaches are faster and may reduce inter-observer inconsistencies [30]. In this regard, artificial intelligence has proved promising in developing models for automated lesion segmentation. In a recent study, Laukamp et al. [13] used a multi-parametric deep learning model for fully automated detection and segmentation of meningiomas, demonstrating a strong correlation with manual segmentation even though MRI images from different scanners were included in the study. In detail, average Dice coefficients were 0.81 ± 0.10 (range: 0.46–0.93) for the total tumor volume, using FLAIR- and contrast enhanced T1-weighted images. Similarly, Chen et al. [34] developed a modified U-Net convolutional neural network segmentation model based on contrast enhanced T1-weighted images, reporting Dice scores of 0.920 ± 0.009 (Figure 1).

As previously stated, reliable lesion segmentation is also crucial before radiation therapy or radiosurgery, important treatment options for meningiomas, to establish the gross target volume. In this setting, Florenz et al. [35] proposed the use of multiparametric MRI texture-based features to improve the differentiation between tumor and edema for gross target volume definition (AUC > 0.71).

## 4. Differential Diagnosis

**Figure 2:**
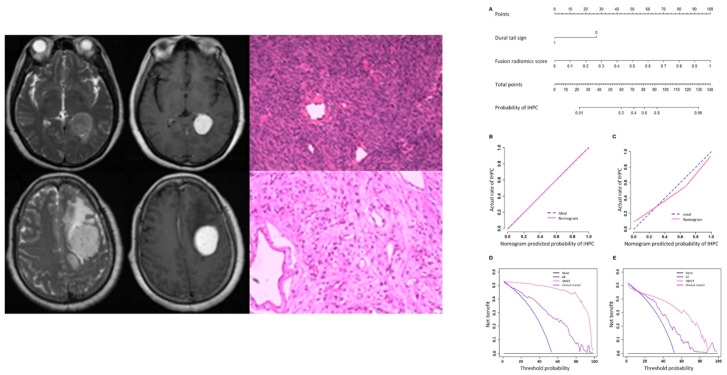
MR and pathological images of a hemangiopericytoma (upper row) and a meningioma (lower row). Nomogram (**A**), calibration curves (**B,C**), and decision analysis curves (**D,E**) in the training and validation cohorts for differential diagnosis between the two conditions. IHPC: intracranial hemangiopericytoma; HMDT: IHPC and Meningioma Diagnostic Tool. Adapted from Ref. [20], under the terms of the Creative Commons Attribution-NonCommercial 4.0 License.

Imaging is generally sufficient to characterize expansive extra-axial lesions such as meningiomas. In some circumstances, however, it may be difficult to distinguish them from other similar neoplasms, in particular solitary fibrous tumor (also known as hemangiopericytoma), very vascularized, potentially causing massive bleeding during surgery, and difficult to differentiate from an angiomatous meningioma on MRI. The differential diagnosis of angiomatous meningioma versus hemangiopericytoma was investigated by Kanazawa et al. [11] They used a texture analysis approach based on contrast enhanced T1- and T2-weighted sequences as well as apparent diffusion coefficient (ADC) maps. The authors showed that ADC entropy and T2 skewness were higher in the case of hemangiopericytoma compared to angiomatous meningioma. Additionally, the mean ADC value was able to differentiate the two lesions with a positive predictive value of 62.5% and specificity of 62.5%. Likewise, Li et al. [14] assessed whether a machine learning model based on texture analysis could allow a differential diagnosis between hemangiopericytoma and angiomatous meningioma. The authors compared clinical and texture features (obtained from FLAIR-, contrast enhanced T1-weighted images and DWI), training four different support vector machine classifiers. They obtained the highest AUC (area under the curve) value (=0.90) when using a contrast enhanced T1-weighted sequence-based classifier. In another study, Wei et al. [20] used a combination of clinical and radiological data to develop an integrated diagnostic tool for preoperative distinction of intracranial hemangiopericytoma from meningioma (Figure 2). This tool demonstrated a remarkable diagnostic accuracy, with an AUC of 0.917 in the validation cohort. Finally, Fan et al. [9] developed a diagnostic model based on a combination of clinical and radiomics features to distinguish the two neoplasms, reporting an AUC of 0.91 in the validation set.

Another relevant differential diagnosis for meningiomas in the sellar region is represented by craniopharyngiomas. In this regard, Tian et al. [19] investigated the ability of qualitative and quantitative MR features in differentiating these two lesions. They found four qualitative and three quantitative parameters, which however could be related to each other, showing significant difference between the two neoplasms. In this context, Zhang et al. [25] investigated the accuracy of machine-learning models in preoperative differentiation of skull base lesions, using both radiomic and clinical features. In the differentiation between meningiomas and craniopharyngiomas the authors obtained an AUC of 0.807 in the testing group, adopting a linear discriminant analysis as the classification algorithm.

## 5. Tumor Consistency

Pre-operative tumor consistency assessment, differentiating soft from firm neoplasms, is of fundamental importance for the neurosurgeon but poorly appraisable with conventional imaging. This information is crucial for surgical strategy and patient treatment, and potentially affects the degree of resection. Indeed, the resection of softer tumors is less challenging for the neurosurgeon, and they have a lower rate of recurrence and morbidity. In particular, soft meningiomas can be removed by means of cutting and suctioning while firm lesions, especially skull base meningiomas, may require additional surgical devices (e.g., ultrasonic aspiration, intraoperative navigation, and electrophysiological monitoring). For this reason, a preoperative imaging-based technique to predict tumor consistency could significantly improve patient management, and several radiomics studies have investigated this topic.

**Figure 3:**
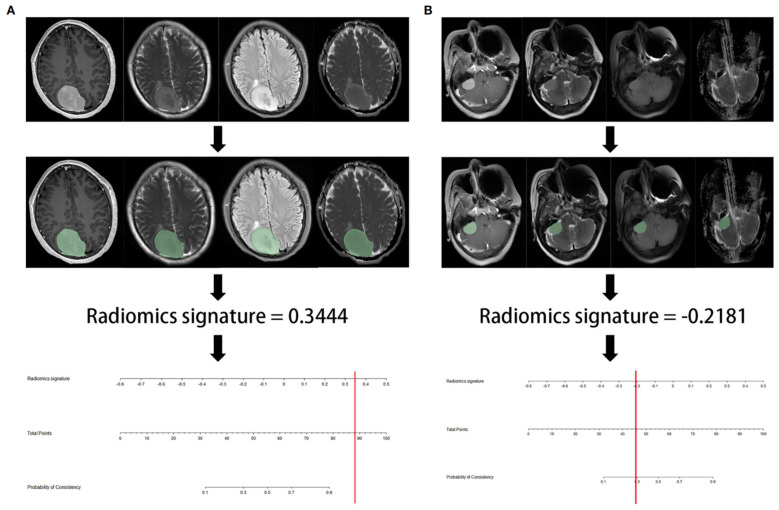
Representative flowchart and radiomics nomogram for meningioma consistency prediction. (**A**) After region of interest delineation, the value of radiomics signature calculated by the algorithm was 0.3444, corresponding to >90% probability of a firm consistency; (**B**) in this case, the radiomics signature was −0.2181, corresponding to a 30% probability of a firm consistency. Consequently, the meningioma consistency was predicted to be soft. In both cases, the predicted consistency was confirmed at surgery. Reproduced from Ref. [23], under the terms of the Creative Commons Attribution-NonCommercial 4.0 License.

AlKubeyyer et al. [4] demonstrated that local binary pattern features from T2-weighted images yielded an AUC of 0.87 when coupled with a k-nearest neighbor classifier for meningioma tumor firmness prediction. In line with this, Zhai et al. [23] validated a radiomics nomogram for predicting meningiomas consistency, based on a logistic regression classifier, which showed an AUC of 0.960 in the test cohort (Figure 3). In a further investigation by Brabec et al. [5], histogram analysis of tensor-valued diffusion MRI has proven promising not only for consistency prediction, but also for grade and type (psammomatous vs. other pooled meningioma types) assessment. Finally, MR-based radiomic features meningiomas have been used to predict consistency estimation obtained with intraoperative ultrasound elastography, with an AUC of 0.96 and classification accuracy of 94% [6].

## 6. Grading

The WHO pathological classification of CNS tumors (4th Edition) stratifies meningiomas in three grades. The majority of these neoplasms are WHO grade I and include meningothelial, fibrous, microcystic, transitional, psammomatous, angiomatous, secretory, metaplastic, and lymphoplasmacyte rich subtypes. Atypical meningiomas, WHO grade II, are defined by 4–19 mitotic figures/10 high-power field or brain invasion or three of these histologic features:

- Increased cellularity;
- Small cells with high N/C ratio;
- Large and prominent nucleoli;
- Patternless or sheet-like growth;
- Foci of “spontaneous” or geographic necrosis.

Furthermore, atypical meningiomas include clear cell and chordoid subtypes.

Finally, WHO grade III anaplastic meningiomas are defined by 20 or more mitotic figures/10 high-power field or sarcomatous or carcinomatous histology and include rhabdoid and papillary subtypes.

Higher-grade meningiomas (WHO grades 2 and 3) are more prone to recurrences, progression, and overall worse prognosis. For this reason, having this information through noninvasive radiomic analysis could be convenient before surgery, to justify a more aggressive surgical approach or to schedule adjuvant radiotherapy after surgery while waiting for pathology report.

The new 5th Edition of the WHO classification of CNS tumors has maintained this grading system but apported some minor changes (e.g., inclusion of TERT promoter mutation among criteria for grade 3 definition) [3]. While these do not currently represent an issue, the natural evolution of grading systems over time in medical imaging and pathology represents a factor to be considered when developing machine learning models. As data labels may change their usefulness or meaning over time, this specific type of data drift is peculiar in the field of medical imaging and will require specific research to address it in the future.

Chen et al. [7] investigated whether texture-analysis-based machine learning algorithms could help identify a non-invasive imaging biomarker for presurgical grading meningiomas from postcontrast T1-weighted images. They obtained an accuracy of 75.6% on the test group using a linear discriminant analysis algorithm. In line with this, Chu et al. [8] obtained an accuracy rate in the test group of 92.9%, with an AUC of 0.948, based on a logistic regression model trained with nine features extracted from contrast-enhanced T1-weighted images. Furthermore, in their multi-center radiomics study based on multiparametric MRI examinations, Hamerla et al. [10] reported an AUC of 0.97 using an XGBoost classifier, despite heterogeneous protocols across different centers. Similarly, Laukamp et al. [36] investigated the role of radiomics-based shape and texture analysis on multiparametric MRI exams from different scanners and institutions for grading meningiomas. In a multivariate logistic regression model, the combination of these features led to an AUC of 0.91.

**Figure 4:**
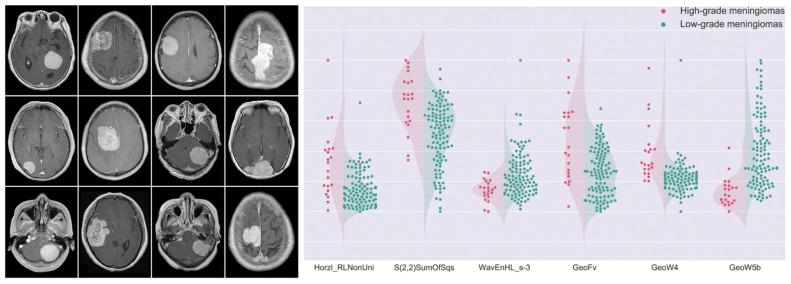
MR images depicting differences in meningioma textural heterogeneity and morphology. The graph presents patient distributions for six radiomics features. High-grade meningiomas are represented in red, while green dots symbolize low-grade lesions. These selected features were significantly different between the two groups. Adapted from Ref. [21], under the terms of the Creative Commons Attribution-NonCommercial 4.0 License.

An important contribution of ADC in meningioma grading has been pointed out by Yiping Lu et al. [15]. In their work, a decision forest classifier, built with 23 selected texture features and the ADC value from the training dataset, achieved an accuracy of 79.51% in the testing cohort. Morin et al. [16] confirmed the value of ADC for this purpose. They investigated prognostic models based on clinical, radiologic, and radiomic features to preoperatively identify meningiomas at risk for poor outcomes, finding that low ADC values were associated with high-grade meningioma, and low sphericity was associated both with increased local failure and worse overall survival. In their work, integrated prognostic models combining clinical, radiologic, and radiomic features demonstrated improved accuracy for meningioma grade, local failure, and overall survival (AUC of 0.78, 0.75, and 0.78, respectively) compared to models based on clinical features alone. Park et al. [17] evaluated the role of radiomics based on postcontrast T1-weighted images, ADC maps, and fractional anisotropy maps, in grading and histological subtyping meningiomas. They achieved an AUC of 0.86 in the validation set. MRI-based radiomics was also used by Yan et al. [21] to predict meningioma grade. They found three texture and three shape features which were significantly different between high-grade and low-grade meningiomas. The SVM classifier achieved the best performance, with an AUC of 0.87 (Figure 4).

A deep learning approach was adopted by Zhu et al. [26], who developed a model based on an improved LeNet-5 model of convolutional neural network using an oversampling technique to address an unbalanced dataset. The accuracy of the model reached a promising value of 83.33% for the classification of meningiomas from MR images. In line with this, a deep learning radiomics model based on routine post-contrast MRI obtained an AUC of 0.811 (Zhu et al. [37]). The authors also demonstrated a superior deep learning performance over typical, hand-crafted features. A further enhanced T1-weighted image-based deep learning model was used by Yang et al. [22] in differentiating low- and high-grade meningiomas. In their study, the combined deep learning–radiomics model outperformed both the deep learning and hand-crafted radiomics models working alone (test AUC: 0.935 vs. 0.918 vs. 0.718).

A recent meta-analysis of machine learning studies for the pre-operative prediction of meningioma grading from brain MRI revealed an overall AUC of 0.88 with a standard error of 0.02. However, low radiomics quality scores of the included studies were reported. For this reason, future studies with higher methodological quality and adequate standardization are necessary before machine learning implementation into clinical practice [38].

## 7. Prediction of Progression and Recurrence

In 1957, a grading scale describing the extent of meningioma resection was introduced by Simpson [39]. This classification, based on residual tumor or infiltrated dura, has demonstrated prognostic significance and for this reason it has been widely used in neurosurgery since its introduction. In particular, a Simpson grade I consists of a complete tumor resection with removal of affected dura and underlying bone. A grade II is defined by tumor resection and coagulation of its dural attachment, while incomplete dural coagulation or bone excision (non-depictable by MRI but based on the neurosurgeon’s opinion) determine a grade III. In Simpson grade IV, a macroscopic residual tumor is visible on MRI. Finally, an incisional biopsy with conspicuous remaining neoplastic tissue determines a grade V.

As is obvious to expect, the presence of residual tumor tissue or infiltrated dura represent risk factors of tumor regrowth. Nevertheless, a subset of meningiomas, even if belonging to Simpson grade I, presents early or, less frequently, delayed progression/recurrence (P/R) after their resection which is very difficult to predict. Conventional MRI findings related to P/R include tumor size, bone invasion, and proximity to the major sinuses, but are unable to accurately predict P/R.

Ko et al. [12] applied pre-operative MR-based radiomics to predict P/R in meningiomas for which a gross total resection was obtained (Simpson grades I–III), employing a support vector machine algorithm. Using the model’s output score, an AUC of 0.80 was obtained for predicting P/R. A shorter progression-free survival was also associated with higher support vector machine scores.

In 20–30% of meningiomas, the lesion originates from the skull base and for this reason it is very challenging to achieve complete surgical resection while avoiding neurological complications, due to the complex neurovascular anatomy of this location. A subtotal tumor resection represents a favorable strategy in these cases but may lead to early P/R. Zhang et al. [24] developed a MR radiomics model showing an accuracy of 90% for P/R prediction in skull base meningiomas undergoing gross-total resection (Simpson grades I–III).

## 8. Prediction of Radiosurgery Response

Complete surgical resection represents the first-line treatment for meningiomas, while stereotactic radiosurgery is reserved for inoperable lesions, representing a complementary or definitive treatment modality for selected patients. In this setting, prediction of volumetric control after stereotactic radiosurgery can provide important information for selecting an adequate treatment strategy. Speckter et al. [18] used a radiomics approach to identify the best feature subsets capable of predicting volumetric changes in meningiomas after treatment with stereotactic radiosurgery (Gamma Knife), using T1- and T2-weighted MR sequences and diffusion tensor imaging (DTI). They demonstrated a higher correlation of meningioma volume reduction with DTI-derived data, followed by T2-weighted image-derived parameters.

## 9. Limitations

Although radiomics analyses are extremely promising in evaluating various aspects of meningiomas, as shown in the previous sections of this review, there are several limitations that also need to be discussed. First of all, the retrospective monocentric design of most current radiomics studies poses several challenges. Such study design could lead to a patient selection bias, with samples having limited representativeness of the general population [40]. Similarly, data in this setting originates from a limited range of equipment vendors and acquisition protocols. Random patterns tied to these could also introduce biases in the models, which can be challenging to detect without access to more varied datasets. In general, these issues may result in models which are not able to reproduce their performance in new clinical settings (i.e., they do not “generalize”). The second issue is partially tied to the prior and consists of the low standardization of imaging protocol standardization in radiology, which could also affect model generalizability and therefore clinical implementation. Novel techniques for data augmentation and harmonization could also be valuable for addressing this issue [41,42,43].

Another concern is represented by relatively small sample sizes in the current literature, potentially leading to overfitting of the prediction model. Further limitations include the extreme heterogeneity of the adopted segmentation, feature extraction, feature selection, and modeling steps. In general, all the mentioned issues represent a potential source of undesirable heterogeneity, originated by patient cohort and imaging data characteristics rather than reflecting underlying biological lesion characteristics. The risk is that radiomics pipelines will detect such heterogeneity as a source of information and incorporate it into the model’s classification process instead of recognizing it for the noise it truly represents and discard it.

In consideration of the aforementioned issues, future efforts should focus on a greater standardization of the radiomics pipeline, as well as on prospective multicentric study designs. Together with this, the creation of high-quality, ideally public, datasets with integration of radiomics, genomics, and proteomics information as well as clinical-prognostic and neuropathological data, could represent the key to success for a real clinical implementation of big data analysis in the field of precision medicine. The benefits that other domains have obtained from such large-scale dataset building efforts [44] indeed seem to support this concept. Even though still in their infancy, efforts are being made both to raise awareness on methodological issues affecting current radiomics research [38,45], raise awareness in editors and reviewers to pose greater attention to technical aspects and clinical relevance of these papers [46,47], increase awareness of potential buyers of commercial solutions based on radiomics [48,49], and collect curated, open medical imaging datasets [50,51].

## 10. Conclusions

Radiomics has the potential to provide valuable information for the management of patients affected by meningioma and many studies have investigated its feasibility in recent years. While some implementations are commercially available, such as for the segmentation of these neoplasms, others, including grading, prognostic and predictive assessment are still far from translation to clinical practice. These models still require more rigorous investigations, especially focused on reproducibility and generalizability of their performance, to allow their inclusion in clinical decision support software and actual improvement of meningioma patient management.

## Figures and Tables

**Table 1:** Overview of meningioma radiomics studies.

Author	Year	Number of Patients	MR Sequences	Aim	Radiomics Analysis	ROI	Outcome
AlKubeyyer et al. [4]	2020	31	T2	Characterization	Machine learning	2D	Tumor firmness
Brabec et al. [5]	2022	30	DTI	Characterization	Histogram analysis	2D	Tumor firmness and presurgical grading
Cepeda et al. [6]	2021	18	CE-T1	Characterization	Machine learning	3D	Tumor firmness
Chen et al. [7]	2019	150	CE-T1	Characterzation	Machine learning	3D	Presurgical grading
Chu et al. [8]	2020	98	CE-T1	Characterization	Machine learning	3D	Presurgical grading
Fan et al. [9]	2022	220	CE-T1, T2	Characterization	Clinic-radiomic model	3D	Differential diagnosis of intracranial hemangiopericytoma and angiomatous meningioma
Hamerla et al. [10]	2019	138	CE-T1, T2, ADC, FLAIR, subtraction maps	Characterization	Machine learning	3D	Presurgical grading
Kanazawa et al. [11]	2018	43	CE-T1, ADC	Characterization	Texture analysis	3D	Differential diagnosis of intracranial hemangiopericytoma and angiomatous meningioma
Ko et al. [12]	2021	128	CE-T1, T2	Prognosis	Radiomic features	3D	Recurrence
Laukamp et al. [13]	2018	211	CE-T1, FLAIR	Segmentation	Deep learning	3D	Segmentation
Li et al. [14]	2019	67	CE-T1, ADC, FLAIR	Characterization	Machine learning	3D	Differential diagnosis of intracranial hemangiopericytoma and angiomatous meningioma
Lu et al. [15]	2018	152	ADC	Detection	Machine learning	3D	Diagnosis
Morin et al. [16]	2019	303	CE-T1	Characterization and prognosis	Radiological-radiomic model	3D	Grading, local failure, survival
Park et al. [17]	2018	136	CE-T1, ADC, DTI	Characterization	Machine learning	3D	Grading and histological type
Speckter et al. [18]	2018	32	CE-T1, T2, T1, DTI	Prognosis	Texture analysis	3D	Treatment response after radiosurgery
Tian et al. [19]	2020	127	CE-T1, T2	Characterization	Texture analysis	3D	Differential diagnosis between craniopharyngioma and meningioma
Wei et al. [20]	2020	292	CE-T1, T2, T1	Characterization	Clinic-radiological data and radiomics signature	3D	Distinction of intracranial hemangiopericytoma from meningioma
Yan et al. [21]	2017	131	CE-T1	Characterization	Machine learning	3D	Presurgical grading
Yang et al. [22]	2022	132	CE-T1	Characterization	Deep learning	3D	Presurgical grading
Zhai et al. [23]	2021	172	CE-T1	Characterization	Machine learning	3D	Meningioma consistency
Zhang et al. [24]	2019	60	T2, ADC	Prognosis	Radiomic classification	3D	Recurrence in skull base meningiomas
Zhang et al. [25]	2020	235	CE-T1	Characterization	Machine learning	3D	Discrimination of lesions located in the anterior skull base
Zhu et al. [26]	2019	222	CE-T1	Characterization	Deep learning	Not reported	Presurgical grading

MR: magnetic resonance; ROI: region of interest; CE: contrast-enhanced; FLAIR: fluid attenuated inversion recovery; ADC: apparent diffusion coefficient; DTI: diffusion tensor imaging.
